# Flexible Data Trimming Improves Performance of Global Machine Learning Methods in Omics-Based Personalized Oncology

**DOI:** 10.3390/ijms21030713

**Published:** 2020-01-22

**Authors:** Victor Tkachev, Maxim Sorokin, Constantin Borisov, Andrew Garazha, Anton Buzdin, Nicolas Borisov

**Affiliations:** 1 OmicsWayCorp, Walnut, CA 91788, USA; tkachev@oncobox.com (V.T.); sorokin@oncobox.com (M.S.); garazha@oncobox.com (A.G.);; 2 Institute for Personailzed Medicine, I.M. Sechenov First Moscow State Medical University, 119991 Moscow, Russia; 3 National Research University—Higher School of Economics, 101000 Moscow, Russia; cortan122@gmail.com; 4 Moscow Institute of Physics and Technology, 141701 Moscow Oblast, Russia; 5 Shemyakin-Ovchinnikov Institute of Bioorganic Chemistry, 117997 Moscow, Russia

**Keywords:** bioinformatics, personalized medicine, oncology, chemotherapy, machine learning, omics profiling

## Abstract

(1) Background: Machine learning (ML) methods are rarely used for an omics-based prescription of cancer drugs, due to shortage of case histories with clinical outcome supplemented by high-throughput molecular data. This causes overtraining and high vulnerability of most ML methods. Recently, we proposed a hybrid global-local approach to ML termed floating window projective separator (FloWPS) that avoids extrapolation in the feature space. Its core property is data trimming, i.e., sample-specific removal of irrelevant features. (2) Methods: Here, we applied FloWPS to seven popular ML methods, including linear SVM, *k* nearest neighbors (kNN), random forest (RF), Tikhonov (ridge) regression (RR), binomial naïve Bayes (BNB), adaptive boosting (ADA) and multi-layer perceptron (MLP). (3) Results: We performed computational experiments for 21 high throughput gene expression datasets (41–235 samples per dataset) totally representing 1778 cancer patients with known responses on chemotherapy treatments. FloWPS essentially improved the classifier quality for all global ML methods (SVM, RF, BNB, ADA, MLP), where the area under the receiver-operator curve (ROC AUC) for the treatment response classifiers increased from 0.61–0.88 range to 0.70–0.94. We tested FloWPS-empowered methods for overtraining by interrogating the importance of different features for different ML methods in the same model datasets. (4) Conclusions: We showed that FloWPS increases the correlation of feature importance between the different ML methods, which indicates its robustness to overtraining. For all the datasets tested, the best performance of FloWPS data trimming was observed for the BNB method, which can be valuable for further building of ML classifiers in personalized oncology.

## 1. Introduction

A personalized approach in oncology was proven helpful for increasing efficacy of drugs prescription in many cancers [1,2]. Generally, it is based on finding specific biomarkers which can be mutations, protein levels or patterns of gene expression [3].

High throughput gene expression data can be connected with responsiveness on treatment using two major approaches. First, drug efficacy can be simulated using hypothesis-driven drug scoring algorithms which utilize knowledge of drugs molecular specificities and up/downregulated statuses of target genes and molecular pathways in a tumor [1,3,4,5,6].

In turn, agnostic drug scoring approach, including machine learning (ML) methods can offer even a wider spectrum of opportunities by non-hypothesis-driven direct linkage of specific molecular features with clinical outcomes, such as responsiveness on certain types of treatment [7,8]. ML has a variety of methods that could be used for such agnostic approach, e.g., decision trees, DT [9,10], random forests, RF [11], linear [12], logistic [13], lasso [14,15], and ridge [16] regressions, multi-layer perceptron, MLP [10,17,18], support vectors machines, SVM [9,10,19], adaptive boosting [20,21,22]. The high throughput transcriptomic data, including microarray- and next-generation sequencing gene expression profiles can be utilized for building such classifiers/predictors of clinical response to a certain type of treatment. However, the direct use of ML to personalize prediction of clinical outcomes is problematic, due to the lack of sufficient amounts of preceding clinically annotated cases supplemented with the high-throughput molecular data (~thousands or tens thousands of cases per treatment scheme) [23]. 

Several ML methods have been recently successfully applied for distinguishing between cancer patients with positive and negative responses on various treatments [20,24,25,26]. However, they were not successful (area under curve (AUC) < 0.66) in predicting clinical outcomes for several model datasets, including multiple myeloma expression dataset associated with known clinical responses on cancer drug bortezomib [20,24,25,26,27].

**Figure 1:**
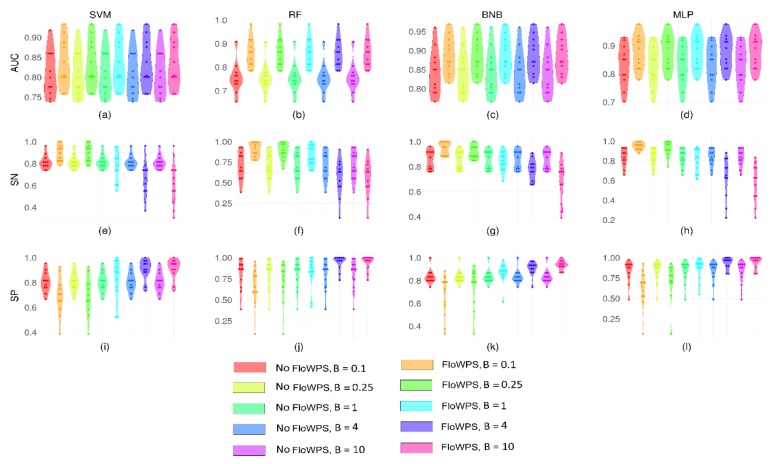
Area under curve (AUC) (**a**–**d**), sensitivity (SN) (**e**–**h**) and specificity (SP) (**i**–**l**) calculated for treatment response classifiers for eleven non-equalized datasets. The classifiers were based on SVM (**a**,**e**,**i**), RF (**b**,**f**,**j**), binomial naïve Bayes (BNB) (**c**,**g**,**k**) and multi-layer perceptron (MLP) (**d**,**h**,**l**) machine learning (ML) methods. The color legend shows the absence or presence of FloWPS in the classifier analytic pipeline and the value of relative balance factor *B*. On each panel, each violin plot shows the distribution of values for eleven cancer datasets.

For the classical ML approaches, most of the clinical genetic datasets are insufficient for effectively solving the task of differentiating treatment responders from non-responders [9,28]. Features measured by sequencing (e.g., polymorphisms, mutations or gene expression values) are far more numerous than the cohorts of individual patients with traced clinical outcomes. For generating statistically significant predictions, extensive reduction of a pool of features under consideration is needed to make their number comparable with the number of individual samples available [10,29,30,31]. To leverage the performance of ML in biomedicine, we recently developed an approach called flexible data trimming (Data trimming (DT) is the process of removing or excluding extreme values, or outliers, from a dataset [32]) [8,29,33,34,35]. This approach is heuristic and based on a common geometrical sense (Figure 1). It utilizes the following basic principles: (i) When a new sample is analyzed to make a prediction, the predictor has to be adapted to a new observation, or re-learned; (ii) the re-learned predictor must be built within a new specific subspace, while using reduced (trimmed) training data.

Excluding non-informative features helps ML classifiers to avoid extrapolation, which is a well-known Achilles heel of ML [36,37,38,39]. Thus, for every point of a *validation* dataset, the *training* dataset is adjusted to form a floating window. We, therefore, called the respective ML approach, floating window projective separator (FloWPS) [8].

In a pilot trial of this approach, it significantly enhanced robustness of the SVM classifier in all ten clinical gene expression datasets totally representing 992 cancer patients either responding or not on the different types of chemotherapy [8]. FloWPS demonstrated surprisingly high performance (the ROC (receiver-operator curve) is a widely used graphical plot that illustrates the diagnostic ability of a binary classifier system as its discrimination threshold is varied. The ROC is created by plotting the true positive rate against the false positive rate at various threshold settings. The area under the ROC curve, called ROC AUC, or simply AUC, is routinely used for assessment of the quality of the classifier. AUC can vary from 0.5 till 1 and the standard threshold discriminating good vs. poor classifiers is AUC > 0.7 or more) of AUC > 0.7 for the leave-one-out scheme in all datasets, including those where responders and non-responders were poorly distinguishable algorithmically in the previous works [20,24,25,26,27]. However, the applicability and usefulness of FloWPS for a wide variety of ML methods remained unstudied. 

Here, we investigated FloWPS performance for seven popular ML methods, including linear SVM, *k* nearest neighbors (kNN), random forest (RF), Tikhonov (ridge) regression (RR), binomial naïve Bayes (BNB), adaptive boosting (ADA) and multi-layer perceptron (MLP). We performed computational experiments for 21 high throughput gene expression datasets (41–235 samples per dataset) corresponding to 1778 cancer patients with known responses on chemotherapy treatments. We showed that FloWPS essentially improved the classifier quality for all *global* ML methods (SVM, RF, BNB, ADA, MLP), where the AUC for the treatment response classifiers increased from 0.65–0.85 range to 0.80–0.95. For all the datasets tested, the best performance of FloWPS data trimming was observed for the BNB method, which can be valuable for further building of ML classifiers in personalized oncology.

Additionally, to test the robustness of FloWPS-empowered ML methods against overtraining, we interrogated agreement/consensus features between the different ML methods tested, which were used for building mathematical models for the classifiers. The lack of such agreement/consensus could indicate overtraining of the ML classifiers built, suggesting random noise instead of extracting significant features distinguishing between the treatment responders and non-responders. If ML methods indeed tend to amplify random noise during overtraining, then one could expect a lack of correlation between the features for geometrically different ML models. However, we found here that (i) there were statistically significant positive correlations between different ML methods in terms of relative feature importance, and (ii) that this correlation was enhanced for the ML methods with FloWPS. We, therefore, conclude that the beneficial role of FloWPS is not due to overtraining. 

## 2. Results

### 2.1. Performance of FloWPS for Equalized Datasets Using All ML Methods with Default Settings

In this study, we used FloWPS in combination with seven ML methods, namely, linear support vector machines (SVM), *k* nearest neighbors (kNN), random forest (RF), ridge regression (RR), binomial naïve Bayes (BNB), adaptive boosting (ADA) and multi-layer perceptron (MLP).

First ten over twenty-one gene expression datasets investigated here had equal numbers of known responders and non-responders and were investigated first. The basic quality characteristics of using seven above ML methods for discriminating between responders and non-responders in these datasets are shown in Supplementary Figures S1_1, S1_2, S1_3, including AUC, sensitivity (SN) and specificity (SP). Each ML method was applied with its default settings using Python package *sklearn* [40], both with and without data trimming, separately for each dataset. Although different values of relative balance factor *B* and discrimination threshold τ (see Materials and Methods, Section 4.3) did not affect the ROC AUC characteristics, they were crucial for sensitivity and specificity (Supplementary Figures S1_1, S1_2, S1_3). 

We found that the use of FloWPS has considerably improved the AUC metric for all global ML methods investigated (SVM, RF, BNB, ADA and MLP), but had no effect on the performance of local methods kNN and RR (Supplementary Figures S1_1, S1_2, S1_3). For the global ML methods, FloWPS improved the classifier quality and increased AUC from 0.61–0.88 range to 0.70–0.94 (Supplementary Figures S1_1, S1_2, S1_3), and AUC median values—from 0.70–0.77 range to 0.76–0.82 (Table 1). In addition, kNN and RR also showed poor SN and SP for *B* > 1 and *B* < 1, respectively (Supplementary Figures S1_1, S1_2, S1_3).

These findings are summarized in Table 1. Considering quality criterion of combining the highest AUC, the highest SN at *B* = 4 and the highest SP at *B* = 0.25, the top three methods identified for the default settings were BNB, MLP and RF (Supplementary Figures S1_1, S1_2, S1_3; Table 1). 

### 2.2. Performance of FloWPS for Equalized Datasets Using BNB, MLP and RF Methods with the Advanced Settings

We then checked the performance of three best ML methods (BNB, MLP and RF) for the same ten datasets with equal numbers of responders and non-responders using advanced settings, see Materials and Methods (Supplementary Figures S2_1, S2_2, S2_3; Table 2). FloWPS improved the classifier quality for these three ML methods and increased AUC from 0.75–0.78 range to 0.83-0.84 (Table 2). 

For RF, the best results were obtained with the following parameter settings: *n_estimators* = 30, *criterion* = “entropy” (Supplementary Figures S2_1, S2_2, S2_3). For BNB, the best settings were *alpha* = 1.0, *binarize* = 0.0, and *fit_prior* = False (Supplementary Figures S2_1, S2_2, S2_3). For MLP, the best settings were *hidden_layer_sizes* = 30, *alpha* = 0.001 (Supplementary Figures S2_1, S2_2, S2_3). Among these three ML methods, the best results were obtained for BNB with *alpha* = 1.0, *binarize* = 0.0, and *fit_prior* = False (Supplementary Figures S2_1, S2_2, S2_3). BNB with these parameter settings can be, therefore, recommended for further development and implementation of the expression-based classifiers of individual treatment response, because it showed simultaneously acceptable AUC, SN and SP for the maximum spectrum of datasets tested (Supplementary Figures S2_1, S2_2, S2_3; Table 2).

### 2.3. Performance of FloWPS for Non-Equalized Datasets Using BNB, MLP, RF and SVM Methods with the Advanced Settings

We then applied the best settings previously found for BNB, MLP and RF methods using responder-equalized data for the new eleven datasets containing different proportions of treatment responders’ and non-responders’ samples. In addition, we also used linear SVM method (Figure 1, Table 3) with penalty parameter *C* = 1 because our previous results [8] showed that *C* ≤ 1 minimizes the risk of overtraining for SVM. The output ML classifier quality metrics were obtained for these four methods, including AUC (Figure 1a–d), SN (Figure 1e–h) and SP (Figure 1i–l). In this trial, the number of responders and non-responder samples were not equal. To compensate for the possible influence of the variable proportion of samples in the two classes, SVM and RF calculations were performed using the *balanced-class* option.

The application of FloWPS improved the classifier quality for these four ML methods, as the median AUC for the treatment response classifiers increased from 0.76–0.84 range to 0.83–0.89 (Figure 1a–d, Table 3). In this experiment, we confirmed the advantage of using FloWPS for all four ML methods tested and the best performance of BNB also for eleven datasets with non-equal numbers of responders and non-responder samples.

### 2.4. Correlation Study Between Different ML Methods at the Level of Feature Importance 

We showed positive pairwise correlations between the different ML methods at the level of relative importance (*I_f_*, see Materials and Methods) of different features tested (Table 4, Supplementary Figures S3_1, S3_2, Supplementary Table S4_1). Greater similarities between *I_f_* marks in the different ML methods reflect more robust applications of the ML. Importantly, the correlations for the ML methods with FloWPS were always higher than for the methods without FloWPS (Table 4, Supplementary Figures S3_1, S3_2). This clearly suggests the beneficial role of FloWPS for extracting informative features from the noisy data. In this model, the biggest similarity was observed for the pair of RR and BNB methods.

## 3. Discussion

**Figure 2:**
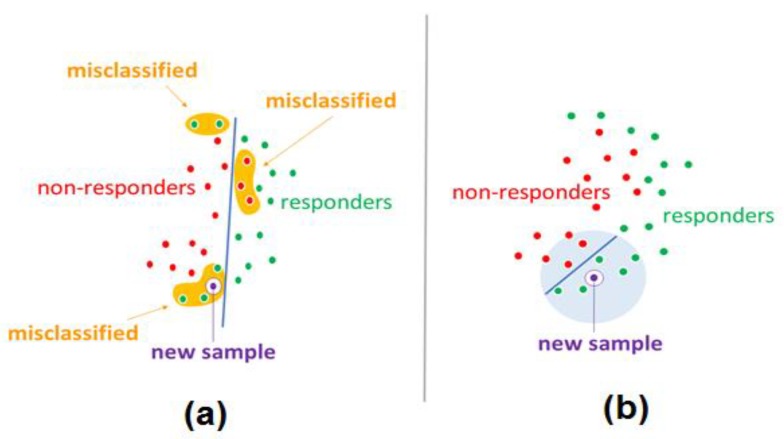
Schematic view of global-local order hybrid ML analytic pipeline (adopted after [8]; copyright belongs to the authors of [8], who wrote also the current paper). (**a**) Global machine learning methods may fail to separate classes for datasets without global order. (**b**) ML, coupled with FloWPS, works locally and handles that cases more accurately.

Many ML methods which were designed for global separation of different classes of points in the feature space are prone to overtraining when the number of preceding cases is low. Global ML methods may also fail if there is only local rather than global order in the placement of different classes in the feature space (Figure 2a).

To improve performance of ML, FloWPS approach includes some elements of the local methods, e.g., using the flexible data trimming that avoids extrapolation in the feature space for each validation point and by selecting only several nearest neighbors from the training dataset. In such a way, the whole ML classifier becomes hybrid, both global and local (Figure 2b).

In this hybrid approach, for each validation point training of ML models is performed in the individually tailored feature space. Every validation point is surrounded by a floating window from the points of the training dataset, and the irrelevant features are avoided using the rectangular projections in the feature space.

This approach was initially tested for the SVM method [8,33,34,35], and in this study, we for the first time applied it to supplement other six popular ML techniques. We used twenty-one clinically annotated gene expression datasets totally, including 1778 patient samples with known clinical treatment responses. These datasets contained 41–235 samples and represented breast cancer (10) multiple myeloma (4), acute myeloid leukemia (3), pediatric acute lymphoblast leukemia (1), pediatric Wilms kidney tumor (1), low grade gliomas (1) and lung cancer (1). The chemotherapeutic treatment schemes included taxanes, bortezomib, vincristine, trastuzumab, letrozole, tipifarnib, temozolomide, busulfan and cyclophosphamide.

We confirmed the efficiency of FloWPS for all tested global ML methods: Linear support vector machines (SVM), random forest (RF), binomial naïve Bayes (BNB), adaptive boosting (ADA) and multi-layer perceptron (MLP). The paired t-test for FloWPS-vs.-no-FloWPS comparison assures that the AUC values for FloWPS-empowered ML methods are significantly higher. For all the datasets tested, the use of FloWPS could increase the quality of binary classifiers for clinical response on chemotherapy.

The regression-like methods, including FloWPS-assisted ML techniques, produce as their outputs the continuous values for likelihood of a sample belonging to a specific class. A discrimination threshold (τ) applied to these output values makes it possible to classify the samples as either responders or non-responders. To set up this threshold, it is important to evaluate the relative penalties of false positive and false negative errors. In most clinically relevant applications, this relative balance factor (*B*) varies between 0.25 and 4 [41,42,43,44,45]. For higher *B* values, the test sensitivity (SN) is low, and lower *B* means lower specificity (SP). 

We found that FloWPS-assisted global ML methods RF, BNB and MLP, exhibited the highest SN and SP in the range 0.25 ≤ *B* ≤ 4 (Supplementary Figures S1_1, S1_2, S1_3; Table 1). Our further and more detailed trial with advanced ML settings confirmed this finding, with the best results shown by the binomial naïve Bayesian (BNB) method with the settings *alpha* = 1.0, *binarize* = 0.0, *fit_prior* = False (Supplementary Figures S2_1, S2_2, S2_3; Table 2). When the best settings identified were applied to eleven cancer datasets with different proportions of the responders and non-responders, FloWPS again was found beneficial for all local ML techniques, and the BNB method showed the best performance (Figure 1c,g,k; Table 3). 

Overtraining, together with extrapolation, is very frequently considered also an Achilles heel of ML. We, therefore, tested if FloWPS helps to extract truly significant features or if it simply adapts to random noise, thus, causing overfitting. We compared four global ML methods (SVM, RF, BNB and MLP) and one local ML method (RR) to check similarities between them in terms of relative importance of distinct individual features. We confirmed that all these five ML methods were positively correlated at the level of feature importance (Table 4, Supplementary Figures S3_1, S3_2). Moreover, using FloWPS significantly enhanced such correlations in all the cases examined (Table 4 Supplementary Figures S3_1, S3_2, Supplementary Table S4_1). These results clearly suggest that FloWPS is helpful for extracting relevant information rather than merely follows the random noise and overfits the ML model.

Overall, we propose that using correlations between different ML methods at the level of relative importance of distinct features may be used as an evaluation metric of ML suitability for building classifiers utilizing omics data (Table 5, Supplementary Figure S5_1). In this case, the higher is the correlation, the bigger should be the probability that the separation of responders from non-responders is robust and non-overtrained.

Surely, very few gene expression/mutation datasets have enough number of clinically annotated preceding cases that are sufficient for building any ML model. For the datasets, which does not have enough cases, the transfer learning approach may be applied. This approach implies that the ML model is trained on a bigger, similar, but quite different, dataset, and then applied to a smaller (validation) dataset. The FloWPS technique has been already tested for transfer learning, and gene expression profiles of cell cultures treated with chemotherapeutic drugs served as training datasets [33,34,35]. Another possibility is to aggregate different smaller datasets into bigger ones. For such aggregation, a new harmonizing technique, which is capable to merge arbitrary number of datasets obtained using arbitrary experimental platforms [46], may be applied.

Of course, transformations in the feature space aimed to adapt it to individual preceding cases is not a new idea in ML [47,48,49]. However, our flexible data trimming approach FloWPS is different because it does not use any pre-selected analytical form of transformation kernels, but instead adapts the feature space aoristically for every particular validation case. The success of using FloWPS for the real-world gene expression datasets, including tens to hundreds of samples prompts further trials of its applicability in biomedicine and in the other fields where increased accuracy of ML classifiers is needed.

## 4. Materials and Methods

### 4.1. Clinically Annotated Molecular Datasets 

We used 21 publicly available datasets, including high throughput gene expression profiles associated with clinical outcomes of the respective patients (Table 6). The biosamples were obtained from tumor biopsies before chemotherapy treatments. The outcomes were response or lack of response on the therapy used, as defined in the original reports (Table 6).

The datasets preparation for the analysis included the following steps [8]:

- Labelling each patient as either *responder* or *non-responder* on the therapy used;
- For each dataset, finding top marker genes having the highest AUC values for distinguishing responder and non-responder classes;
- Performing the leave-one-out (LOO) cross-validation procedure to complete the robust core marker gene set used for building the ML model.

### 4.2. Principles of Flexible Data Trimming

We first introduced [33,34,35] flexible data trimming as a preprocessing tool for transferring to real patients the gene expression data obtained for cell cultures treated with anti-cancer drugs.

Then this method was overhauled and used to increase the SVM-based classifier’s performance for the datasets that contained only gene expression data for cancer patients [8,29]. Since the number of patients with annotated case histories (when treatment method and its clinical success is known, together with the high-throughput gene expression/mutation profile) is limited, we have tailored the whole data trimming scheme to match the leave-one-out (LOO) methodology. 

This LOO approach in our method is employed three times [8,29]:

- First, it helped us to specify the *core marker gene sets* (see Materials and Methods), which form the feature space **F** = (*f*_1_,…,*f_S_*) for subsequent application of data trimming;
- Second, it was applied for every ML prediction act for the wide range of data trimming parameters, *m* and *k*;
- Third, it was used for the final prediction of the treatment response for every patient and optimized (for all remaining patients) values of parameters *m* and *k*.

**Figure 3:**
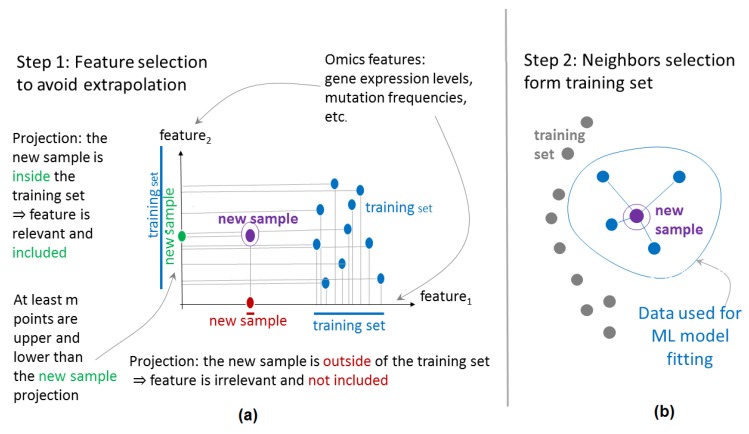
Outline of floating window projective separator (FloWPS) approach. Selection of relevant features (**a**) and nearest neighbors (**b**) are schematized.

Now let us describe flexible data trimming in more detail. Imagine that we have to classify the clinical response for a certain patient *I* (called *patient of interest)* from a given dataset. Let the whole dataset contain *N* patients, so that the remaining *N* − 1 patients form the *preceding dataset D_i_*, for the patient of interest. For ML *without data trimming*, in the feature space **F** = (*f*_1_,…,*f_S_*) all *N – 1* remaining patients are used to build the classifier. However, in the case of FloWPS, LOO procedure will be applied to classify every sample *j ≠ i* from the preceding dataset *D_i_* without sample *i*, and *N − 2* remaining samples may be used for such a classification of sample *j*. To avoid extrapolation in the feature space, we consider the subset **F***_ij_* of *relevant features* [8]. A feature *f_s_* is considered relevant for the sample *j* if on its axis there are at least *m* projections from *N − 2* training samples, which are larger than *f_s_*(*i,j*), and, at the same time, at least *m*, which are smaller than *f_s_*(*i,j*), when *m* is a non-negative integer parameter (Figure 3a). The maximum possible *m* value is (*N – 2*)/2, since if *m* is less than (*N – 2*)/2, then no relevant features may be chosen. Similarly, the minimal case of *m* = 0 also corresponds to no feature selection. Note that the resulting subset of relevant features **F***_ij_*(*m*) will be individual for every pair of samples *i* and *j* [8].

Moreover, in the space **F***_ij_* (*m*) only *k* closest samples to sample *j* will be allowed for training among the remaining (*N – 2*) cases. As a measure for proximity, the Euclidean distance is used [8]. Here *k* is another integer parameter that specifies the number of nearest neighbors in the subspace of selected features (Figure 3b). The maximal possible *k* is *N – 2*, which corresponds to no training sample selection. In contrast, when *k* is too low, there is an increased risk of ML error, due to the presence of a too-small number of training points among the *k* nearest neighbors (Figure 3b). 

After selection of relevant features and nearest neighbors for the sample *j*, the ML model is trained using nearest neighbors only, and used for prediction of a clinical response, *P_ij_*(*m*,*k*), for the patient *j*. After repeating this procedure for all other *j ≠ i*, we obtain the area-under the ROC curve, AUC*_i_* (*m*,*k*), for all, but *i*-th samples for fixed values of data trimming parameters *m* and *k*. 

The AUC_i_ (m,k) can be then analyzed as a function of m and k [8]. Over the range of possible m and *k* values, we compare the AUC_i_ function [8]. All pairs of (m,k) values that provide AUC_i_ (m,k) > *p*·max (AUC_i_ (m,k)) form the prediction-accountable set S_i_ for the patient of interest *i* [8], where *p* is the confidence threshold, which could vary from 0.90 till 0.95 in our previous computational experiments [8].

Finally, the FloWPS prediction *P_Fi_* for the sample of interest *i*, is calculated by averaging the ML predictions over the prediction-accountable set *S_i_*: $P_{Fi}=mean_{Si}\left(P_{i}\left(m,k\right)\right)$. By repeating this procedure for all other samples, a set of FloWPS predictions will be obtained for the whole dataset [8]. 

**Figure 4:**
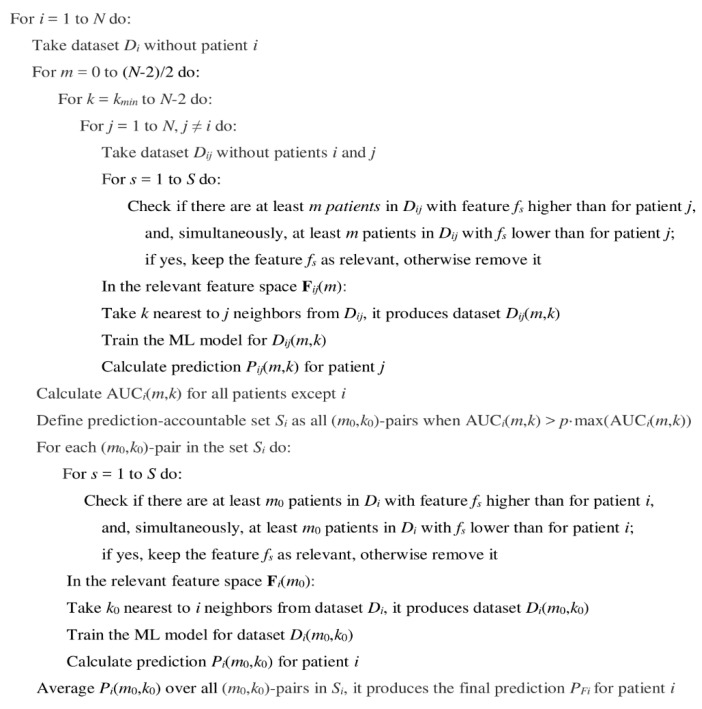
The algorithm of data trimming used for binomial naïve Bayes (LOO) cross-validation of the clinically annotated gene expression datasets. Indexes *i* and *j* denote samples (patients), index *s* denotes pairs of (*m*_0_,*k*_0_)-values in the prediction-accountable set, and indexes *m* and *k* denote the data trimming parameters.

The overview of LOO cross-validation algorithm for FloWPS-empowered ML-based predictor is shown in Figure 4.

The application of ML methods *without FloWPS* means that prediction is made for each sample *i* using the parameter values *m* = 0, *k* = *N − 1*, and a training dataset *D_i_* (without sample *i*).

### 4.3. Application of ML Methods

All the ML calculations were performed using our R package flowpspkg.tar.gz, ffsdf available at Gitlab through the link: https://gitlab.com/borisov_oncobox/flowpspkg. This package, which was prepared for convenience of R users, is a wrapper over a Python code, which is also runnable. The Python code is based on library *sklearn* [40].

For the default settings trial, linear support vector machines (SVM), *k* nearest neighbors (kNN), random forest (RF), ridge regression (RR), binomial naïve Bayes (BNB), adaptive boosting (ADA) and multi-layer perceptron (MLP) were used with the *default parameter settings* for the *sklearn* package. For the advanced settings trial, three ML methods, which showed the best sensitivity and specificity for default settings within the range of relative balance factor 0.25 ≤ *B* ≤ 4, were run under the following conditions. For RF, the parameter *n_estimators* = 10, 30 or 100, and *criterion* = “gini” or “entropy” were used (totally 3 × 2 = 6 setting cases). For BNB, the parameters *alpha* = 0.0 or 1.0, *binarize* = 0.0 or 1.0, and *fit_prior* = True or False, were tried (totally 2 × 2 × 2 = 8 setting cases). For MLP, the parameters *hidden_layer_sizes* = 30 or 100, and *alpha* = 0.01, 0.001 or 0.0001 were checked (totally 2 × 3 = 6 setting cases). For the datasets with an unequal number of responders and non-responder samples (Table 6), linear SVM and RF calculations were done with setting *class_weight = “balanced”* and *class_weight = “balanced_subsample”*, respectively. All other parameters were used with the default settings.

### 4.4. False Positive Vs. False Negative Error Balance

For all ML methods, the FloWPS predictions (*P_Fi_*) were made which were likelihoods for attribution of samples to one of the two classes (clinical responders or non-responders).

The *discrimination threshold* (τ), which may be applied to distinguish between the two classes, should be determined according to the cost balance between false positive (FP) and false negative (FN) errors. In our previous study [8], for determination of the τ value, we considered the costs for FP and FN errors to be equal, and then maximized the overall accuracy rate, ACC = (TP + TN)/(TP + TN + FP + FN), since the class sizes were equal.

In a more general case, the penalty value *p* = *B*·FP + FN is minimized; here, *B* is called relative balance factor. *B* is less than 1 for the situations when the FN error (e.g., refusal of prescription of a drug which might help the patient) is more dangerous than the FP error (e.g., prescription of a useless treatment). Contrary, *B* is greater than 1, when it is safer not to prescribe treatment for a patient than to prescribe it. Several practitioners of clinical diagnostic tests have different opinions on how high/low should be this balance factor. In different applications, the preferred values can be *B* = 4 [41,42,45], *B* < 0.16 [70], 4.5 < *B* < 5 [44], *B* < 5 [43], *B* > 10 for emergency medicine only [71], *B* > 5 for toxicology [72]. 

In case of oncological disease, *B* should be low when only *one or few* treatment options is/are available for a certain patient, because the refusal to give a treatment may cause serious harm to the patient. Contrarily, in the situation when the best treatment plan must be selected among *multiple* options available, the risk of wrong drug prescription will be higher, and *B* should be high as well. For our analyses, we used five model settings of *B* equal to 0.1, 0.25, 1, 4 or 10.

### 4.5. Feature Importance Analysis

For linear SVM, RF, RR, BNB and MLP methods and for all transcriptomic datasets tested, we calculated *relative importance*, *I_f_*, of each gene expression feature *f* in the dataset, using the following attributes of ML classes in Python library *sklearn* [40]:

For linear SVM: *I_f_* = |*coef*_[0]*_f_*|, where *coef*_[0] is the normal vector to the separation hyperplane between responders and non-responders in the feature space in the training model.

For RF, *I_f_* = |*feature_importances_f_*| from the training model.

For RR, $I_{f}=\underset{t}{\sum }\left|X\_fit_{tf}\right|$ , where the summation runs through every sample *t* in the training model.

For BNB,$I_{f}=\;\underset{c}{\sum }feature\_count_{cf}$, where the values named *feature_count_cf_* denote the number of samples encountered for each class *c* and feature *f* during fitting of the training model.

For MLP, $I_{f}=\underset{t}{\sum }\left|coefs{\left[0\right]}_{tf}\right|$, where $coefs{\left[0\right]}_{tf}$ is the coefficient matrix in the first layer of the neural network for feature *f* of sample *t* in the training model. 

For each validation point *I*, the *I_f_* was averaged over all predication-accountable set *S_i_*.

## 5. Conclusions

We applied a flexible data trimming technique FloWPS to enhance performance of seven popular ML methods, including linear SVM, *k* nearest neighbors (kNN), random forest (RF), Tikhonov (ridge) regression (RR), binomial naïve Bayes (BNB), adaptive boosting (ADA) and multi-layer perceptron (MLP). We performed computational experiments for 21 high throughput gene expression datasets (41–235 samples per dataset) totally, including 1778 cancer patients with known responses on chemotherapy treatments. FloWPS essentially improved the classifier quality for all global ML methods (SVM, RF, BNB, ADA, MLP), where the area under the receiver-operator curve (ROC AUC) for the treatment response classifiers increased from 0.61–0.88 range to 0.70–0.94. The comparison of five best ML methods (SVM, RF, RR, BNB and MLP) at the level of relative importance for different features confirmed that ML models used here were not overtrained and that the usage of FloWPS increased the correlations between the different ML methods at the level of feature importance. For all the datasets tested, the best performance of FloWPS data trimming was observed for the BNB method, which can be valuable for further building of ML classifiers in personalized oncology.

## Figures and Tables

**Table 1:** Performance metrics for seven ML methods with default settings for datasets with equal numbers of responders and non-responders.

ML Method	Method Type	Median AUC without FloWPS	Median AUC with FloWPS	Paired *t*-Test *p*-Value for AUC with-vs.-w/o FloWPS	Advantage of FloWPS	Median SN at *B* = 4	Median SP at *B* = 0.25
SVM	Global	0.74	0.80	1.3 × 10^−5^	Yes	0.45	0.42
kNN	Local	0.76	0.75	0.53	No	0.25	0.34
RF	Global	0.74	0.82	1.3 × 10^-5^	Yes	0.45	0.42
RR	Local	0.80	0.79	0.16	No	0.36	0.41
BNB	Global	0.77	0.82	2.7 × 10^−4^	Yes	0.51	0.58
ADA	Global	0.70	0.76	2.4 × 10^−4^	Yes	0.32	0.41
MLP	Global	0.73	0.82	6.4 × 10^−5^	Yes	0.53	0.53

Yes–FloWPS is beneficial for ML quality, No–FloWPS is not beneficial for ML quality.

**Table 2:** Performance metrics for BNB, MLP and RF methods with the advanced settings for datasets with equal numbers of responders and non-responder samples.

ML Method	Median AUC without FloWPS	Median AUC with FloWPS	Paired *t*-Test *p*-Value for AUC with-vs.-w/o FloWPS	Median SN at *B* = 4	Median SP at *B* = 0.25
RF	0.75	0.83	3.5 × 10^−6^	0.50	0.56
BNB	0.78	0.83	6.7 × 10^−4^	0.50	0.60
MLP	0.77	0.84	2.4 × 10^−4^	0.50	0.51

**Table 3:** Performance metrics for BNB, MLP, RF and SVM methods with the advanced settings for eleven datasets with variable numbers of responders and non-responder samples.

Method	Median AUC without FloWPS	Median AUC with FloWPS	Paired *t*-Test *p*-Value for AUC with-vs.-w/o FloWPS	Median SN at *B* = 4	Median SP at *B* = 0.25
SVM	0.81	0.83	0.013	0.65	0.70
RF	0.76	0.86	4.9 × 10^−6^	0.56	0.71
BNB	0.84	0.89	7.5 × 10^−4^	0.78	0.75
MLP	0.83	0.88	1.0 × 10^−4^	0.63	0.71

**Table 4:** Median pairwise Pearson/Spearman correlation at feature (gene expression) importance (*I_f_*) level. Figures above main diagonal: With FloWPS; figures below: Without FloWPS.

	SVM	RF	RR	BNB	MLP
SVM	1	0.53/0.55	0.40/0.39	0.37/0.34	0.46/0.46
RF	0.34/0.40	1	0.51/0.32	0.48/0.31	0.59/0.38
RR	0.19/0.14	0.35/0.04	1	0.93/0.79	0.89/0.52
BNB	0.24/0.14	0.33/0.09	0.88/0.64	1	0.81/0.46
MLP	0.33/0.30	0.40/0.17	0.76/0.06	0.61/0.12	1

**Table 5:** Minimal, median, mean and maximal Pearson/Spearman correlation values for pairwise comparison of different ML methods with FloWPS at the level of feature importance (*I_f_*).

Dataset #	Dataset ID	Min	Median	Mean	Max
1	GSE25066	0.41/0.28	0.72/0.44	0.67/0.46	0.93/0.81
2	GSE41998	−0.02/−0.10	0.55/0.39	0.49/0.35	0.87/0.83
3	GSE9782	0.37/0.19	0.58/0.41	0.62/0.41	0.97/0.88
4	GSE39754	0.34/0.28	0.50/0.37	0.54/0.41	0.84/0.72
5	GSE68871	0.50/0.43	0.62/0.60	0.68/0.64	0.95/0.93
6	GSE55145	0.32/0.29	0.57/0.42	0.60/0.45	0.85/0.70
7	TARGET50	0.34/0.57	0.69/0.74	0.66/0.72	0.95/0.82
8	TARGET10	0.32/0.30	0.50/0.45	0.58/0.48	0.90/0.77
9	TARGET20 busulfan	0.63/0.55	0.70/0.66	0.76/0.70	0.97/0.89
10	TARGET20 no busulfan	0.16/0.35	0.63/0.53	0.60/0.55	0.92/0.79
11	GSE18728	0.38/0.21	0.54/0.46	0.62/0.45	0.95/0.79
12	GSE20181	0.33/0.17	0.43/0.43	0.56/0.43	0.96/0.79
13	GSE20194	0.06/0.04	0.50/0.30	0.49/0.34	0.93/0.80
14	GSE23988	0.28/0.18	0.46/0.35	0.55/0.39	0.96/0.82
15	GSE32646	0.23/0.11	0.37/0.28	0.49/0.32	0.95/0.74
16	GSE37946	0.40/0.26	0.62/0.45	0.62/0.44	0.92/0.69
17	GSE42822	0.34/0.03	0.52/0.40	0.58/0.38	0.89/0.82
18	GSE5122	0.12/−0.06	0.40/0.20	0.46/0.25	0.93/0.79
19	GSE59515	0.37/0.26	0.47/0.47	0.59/0.49	0.96/0.74
20	TCGA-LGG	0.27/0.13	0.64/0.47	0.63/0.42	0.94/0.76
21	TCGA-LC	0.44/0.23	0.62/0.55	0.66/0.53	0.95/0.90

**Table 6:** Clinically annotated gene expression datasets used in this study.

Reference	Dataset ID	Disease Type	Treatment	Experimental Platform	Number NC of Cases (R vs. NR)	Number S of Core Marker Genes
[50,51]	GSE25066	Breast cancer with different hormonal and HER2 status	Neoadjuvant taxane + anthracycline	Affymetrix Human Genome U133 Array	235 (118 R: Complete response + partial response; 117 NR: Residual disease + progressive disease)	20
[52]	GSE41998	Breast cancer with different hormonal and HER2 status	Neoadjuvant doxorubicin + cyclophosphamide, followed by paclitaxel	Affymetrix Human Genome U133 Array	68 (34 R: Complete response + partial response; 34 NR: Residual disease + progressive disease)	11
[27]	GSE9782	Multiple myeloma	Bortezomib monotherapy	Affymetrix Human Genome U133 Array	169 (85 R: Complete response + partial response; 84 NR: No change + progressive disease)	18
[53]	GSE39754	Multiple myeloma	Vincristine + adriamycin + dexamethasone followed by autologous stem cell transplantation (ASCT)	Affymetrix Human Exon 1.0 ST Array	124 (62 R: Complete, near-complete and very good partial responders, 62 NR: Partial, minor and worse)	16
[54]	GSE68871	Multiple myeloma	Bortezomib-thalido-mide-dexamethasone	Affymetrix Human Genome U133 Plus	98 (49 R: Complete, near-complete and very good partial responders, 49 NR: Partial, minor and worse)	12
[55]	GSE55145	Multiple myeloma	Bortezomib followed by ASCT	Affymetrix Human Exon 1.0 ST Array	56 (28 R: Complete, near-complete and very good partial responders, 28 NR: Partial, minor and worse)	14
[56,57]	TARGET-50	Pediatric kidney Wilms tumor	Vincristine sulfate + cyclosporine, cytarabine, daunorubicin + conventional surgery + radiation therapy	Illumina HiSeq 2000	72 (36 R, 36 NR: See Reference [8])	14
[56,58]	TARGET-10	Pediatric acute lymphoblastic leukemia	Vincristine sulfate + carboplatin, cyclophosphamide, doxorubicin	Illumina HiSeq 2000	60 (30 R, 30 NR: See Reference [8])	14
[56]	TARGET-20	Pediatric acute myeloid leukemia	Non-target drugs (asparaginase, cyclosporine, cytarabine, daunorubicin, etoposide; methotrexate, mitoxantrone), including busulfan and cyclophosphamide	Illumina HiSeq 2000	46 (23 R, 23 NR: See Reference [8])	10
[56]	TARGET-20	Pediatric acute myeloid leukemia	Same non-target drugs, but excluding busulfan and cyclophosphamide	Illumina HiSeq 2000	124 (62 R, 62 NR: See Reference [8])	16
[59]	GSE18728	Breast cancer	Docetaxel, capecitabine	Affymetrix Human Genome U133 Plus 2.0 Array	61 (23R: Complete response + partial response; 38 NR: Residual disease + progressive disease)	16
[60,61]	GSE20181	Breast cancer	Letrozole	Affymetrix Human Genome U133A Array	52 (37 R: Complete response + partial response; 15 NR: Residual disease + progressive disease)	11
[62]	GSE20194	Breast cancer	Paclitaxel; (tri)fluoroacetyl chloride; 5-fluorouracil, epirubicin, cyclophosphamide	Affymetrix Human Genome U133A Array	52 (11 R: Complete response + partial response; 41 NR: Residual disease + progressive disease)	10
[63]	GSE23988	Breast cancer	Docetaxel, capecitabine	Affymetrix Human Genome U133A Array	61 (20 R: Complete response + partial response; 41 NR: Residual disease + progressive disease)	18
[64]	GSE32646	Breast cancer	Paclitaxel, 5-fluorouracil, epirubicin, cyclophosphamide	Affymetrix Human Genome U133 Plus 2.0 Array	115 (27 R: Complete response + partial response; 88 NR: Residual disease + progressive disease)	17
[65]	GSE37946	Breast cancer	Trastuzumab	Affymetrix Human Genome U133A Array	50 (27 R: Complete response + partial response; 23 NR: Residual disease + progressive disease)	14
[66]	GSE42822	Breast cancer	Docetaxel, 5-fluorouracil, epirubicin, cyclophosphamide, capecitabine	Affymetrix Human Genome U133A Array	91 (38 R: Complete response + partial response; 53 NR: Residual disease + progressive disease)	13
[67]	GSE5122	Acute myeloid leukemia	Tipifarnib	Affymetrix Human Genome U133A Array	57 (13 R: Complete response + partial response + stable disease; 44 R: Progressive disease)	10
[68]	GSE59515	Breast cancer	Letrozole	Illumina HumanHT-12 V4.0 expression beadchip	75 (51 R: Complete response + partial response; 24 NR: Residual disease + progressive disease)	15
[69]	TCGA-LGG	Low-grade glioma	Temozolomide + (optionally) mibefradil	Illumina HiSeq 2000	131 (100 R: Complete response + partial response + stable disease; 31 NR: Progressive disease)	9
[69]	TCGA-LC	Lung cancer	Paclitaxel + (optionally),cisplatin/carboplatin, reolysin	Illumina HiSeq 2000	41 (24 R: Complete response + partial response + stable disease; 17 NR: Progressive disease)	7

## Supplementary Materials

The following are available online at https://www.mdpi.com/1422-0067/21/3/713/s1, Figure S1_1: Area under the receiver-operator curve (ROC AUC) for treatment response classifiers for ten cancer datasets (see Table 1). The classifiers were based on SVM (A), kNN (B), RF (C), RR (D), BNB (E), ADA (F) and MLP (G) with default in-built parameter settings according to the Python package *sklearn* [40]. The color legend shows the absence or presence of FloWPS in the classifier analytic pipeline and the value of relative balance factor *B*. On each panel, each violin plot shows the distribution of values for eleven cancer datasets, Figure S1_2: Sensitivity (SN) for treatment response classifiers for ten cancer datasets (see Table 1). The classifiers were based on SVM (A), kNN (B), RF (C), RR (D), BNB (E), ADA (F) and MLP (G) with default in-built parameter settings according to the Python package *sklearn* [40] The color legend shows the absence or presence of FloWPS in the classifier analytic pipeline and the value of relative balance factor *B*. On each panel, each violin plot shows the distribution of values for eleven cancer datasets, Figure S1_3: Specificity (SP) for treatment response classifiers for ten cancer datasets (see Table 1). The classifiers were based on SVM (A), kNN (B), RF (C), RR (D), BNB (E), ADA (F) and MLP (G) with default in-built parameter settings according to the Python package *sklearn* [40]. The color legend shows the absence or presence of FloWPS in the classifier analytic pipeline and the value of relative balance factor *B*. On each panel, each violin plot shows the distribution of values for eleven cancer datasets, Figure S2_1: Area under the receiver-operator curve (ROC AUC) for treatment response classifiers for ten cancer datasets (see Table 1). The classifiers were based on RF (A), BNB (B), and MLP (C) with the best settings, as well as RF (D), BNB (E), and MLP (F) with default settings, and RF (G), BNB (H), and MLP (I) with worst settings in the Python package *sklearn* [40] The color legend shows the absence or presence of FloWPS in the classifier analytic pipeline and the value of relative balance factor *B*. On each panel, each violin plot shows the distribution of values for eleven cancer datasets, Figure S2_2: Sensitivity (SN) for treatment response classifiers for ten cancer datasets (see Table 1). The classifiers were based on RF (A), BNB (B), and MLP (C) with the best settings, as well as RF (D), BNB (E), and MLP (F) with the default settings, and RF (G), BNB (H), and MLP (I) with worst settings in the Python package *sklearn* [40] The color legend shows the absence or presence of FloWPS in the classifier analytic pipeline and the value of relative balance factor *B*. On each panel, each violin plot shows the distribution of values for eleven cancer datasets, Figure S2_3: Specificity (SP) for treatment response classifiers for ten cancer datasets (see Table 1). The classifiers were based on RF (A), BNB (B), and MLP (C) with the best settings, as well as RF (D), BNB (E), and MLP (F) with default settings, and RF (G), BNB (H), and MLP (I) with worst settings in the Python package *sklearn* [40]. The color legend shows the absence or presence of FloWPS in the classifier analytic pipeline and the value of relative balance factor *B*. On each panel, each violin plot shows the distribution of values for eleven cancer datasets, Figure S3_1: Pairwise correlations (red—Pearson, green—Spearman) at feature (gene expression) level between different ML methods: Figures above the main diagonal—with FloWPS, figures below—without FloWPS, Figure S3_2: Overall pairwise correlations as similarity metric for each ML method at feature (gene expression) level: A—Pearson, FloWPS, B—Pearson, no FloWPS, C—Spearman, FloWPS, D—Spearman, no FloWPS, Figure S5_1: Correlation values between different ML methods at feature (gene expression) level for each dataset; dataset numbers (see Table 5 in the Main text) are shown through the horizontal axis). A—Pearson, FloWPS, B—Pearson, no FloWPS, C—Spearman, FloWPS, D—Spearman, no FloWPS. Table S4_1: Paired *t*-test *p*-value for FloWPS-vs.-no-FloWPS comparison of correlation coefficients between feature importance for the same datasets. Figures above the main diagonal: Comparison of Pearson correlation coefficients. Figures below the main diagonal: Comparison of Spearman correlation coefficients.

## Abbreviations

ADA	Adaptive boosting
AML	Acute myelogenous leukemia
ASCT	Allogeneic stem cell transplantation
AUC	Area under curve
BNB	Binomial naïve Bayes
FloWPS	Floating window projective separator
FN	False negative
FP	False positive
GEO	Gene expression omnibus
GSE	GEO series
HER2	Human epidermal growth factor receptor 2
kNN	*k* nearest neighbors
LOO	Leave-one-out
ML	Machine learning
MLP	Multi-layer perceptron
RF	Random forest
ROC	Receiver operating characteristic
RR	Ridge regression
SN	Sensitivity
SP	Specificity
SVM	Support vector machine
TP	True positive
TN	True negative
