# Supplementary material for: Flexible Data Trimming Improves Performance of Global Machine Learning Methods in Omics-Based Personalized Oncology
Source: Int J Mol Sci. 2020 Jan 22;21(3):713. doi: 10.3390/ijms21030713 (PMC7037338; doi:10.3390/ijms21030713)
Supplement: Supplementary file 1 [file ijms-21-00713-s001.zip › Suppl_4.docx]

Table S4_1. Paired t-test p-value for FloWPS-vs-no-FloWPS comparison of correlation coefficients between feature importance for the same datasets. Figures above the main diagonal: comparison of Pearson correlation coefficients. Figures below the main diagonal: comparison of Spearman correlation coefficients.

|  | SVM | RF | RR | BNB | MLP |
| --- | --- | --- | --- | --- | --- |
| SVM | 1 | 1.6E-05 | 6.0E-06 | 4.5E-06 | 4.4E-07 |
| RF | 8.8E-05 | 1 | 1.1E-04 | 2.2E-06 | 5.9E-07 |
| RR | 7.3E-06 | 5.0E-07 | 1 | 9.6E-04 | 1.6E-04 |
| BNB | 1.5E-06 | 1.4E-08 | 3.6E-05 | 1 | 7.1E-07 |
| MLP | 1.9E-06 | 1.3E-08 | 3.2E-09 | 1.9E-07 | 1 |
