# Supplementary material for: Flexible Data Trimming Improves Performance of Global Machine Learning Methods in Omics-Based Personalized Oncology
Source: Int J Mol Sci. 2020 Jan 22;21(3):713. doi: 10.3390/ijms21030713 (PMC7037338; doi:10.3390/ijms21030713)
Supplement: Supplementary file 1 [file ijms-21-00713-s001.zip › Suppl_5.docx]

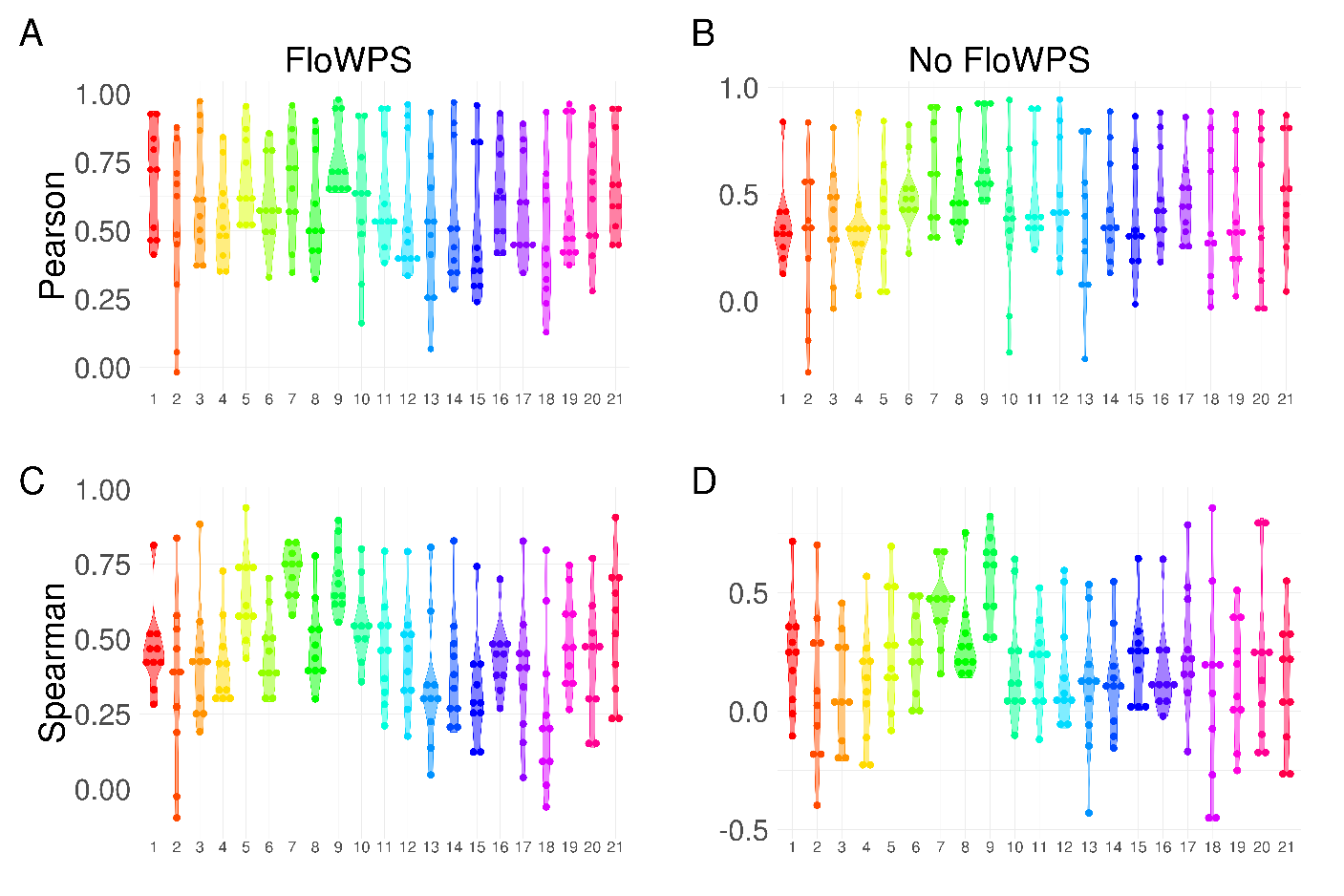


Fig. S5_1. Correlation values between different ML methods at feature (gene expression) level for each dataset; dataset numbers (see Table 5 in the Main text) are shown thorough the horizontal axis). A – Pearson, FloWPS, B – Pearson, no FloWPS, C – Spearman, FloWPS, D – Spearman, no FloWPS.
