# Supplementary material for: Flexible Data Trimming Improves Performance of Global Machine Learning Methods in Omics-Based Personalized Oncology
Source: Int J Mol Sci. 2020 Jan 22;21(3):713. doi: 10.3390/ijms21030713 (PMC7037338; doi:10.3390/ijms21030713)
Supplement: Supplementary file 1 [file ijms-21-00713-s001.zip › Suppl_3.docx]

**
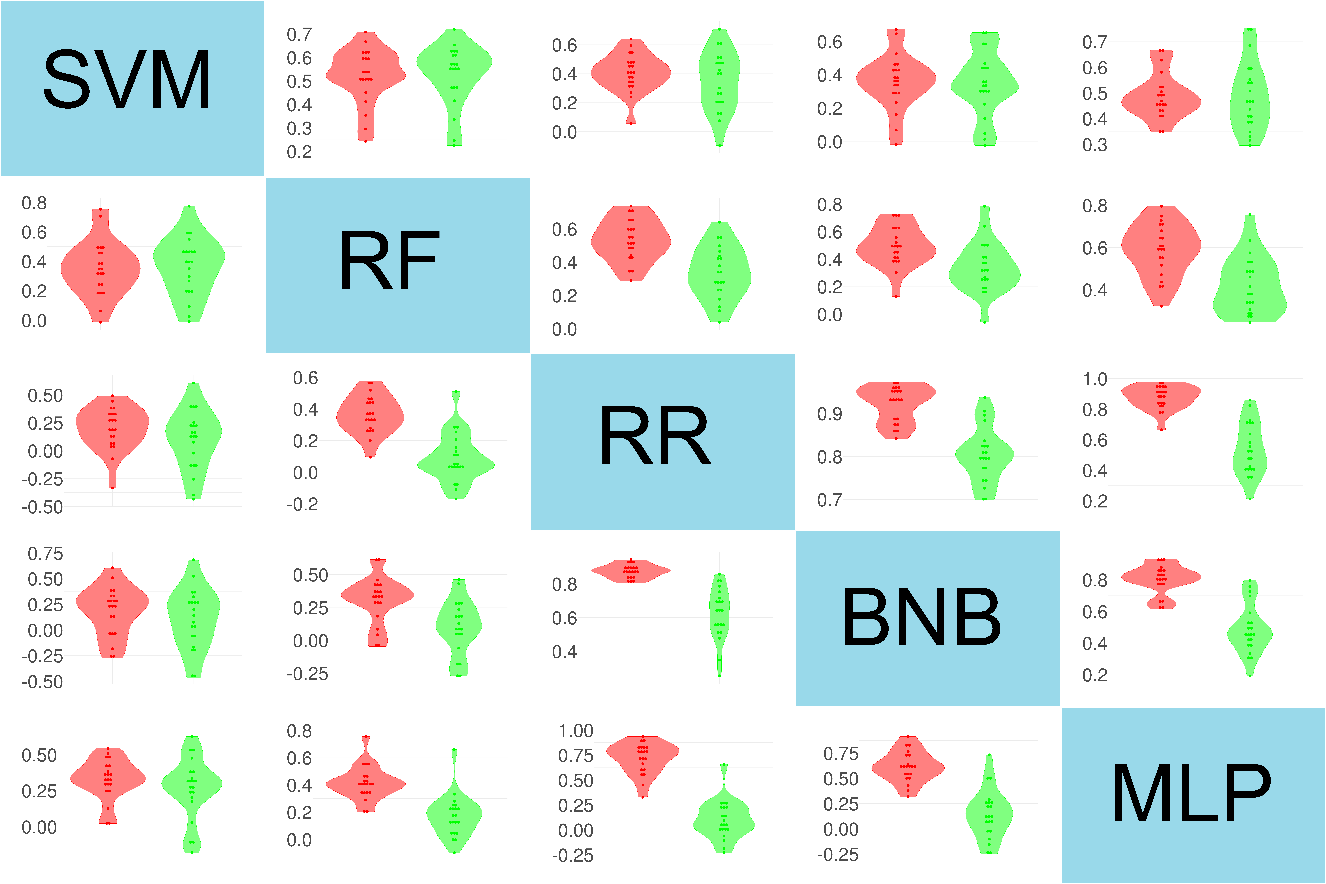
**

Fig. S3_1. Pairwise correlations (red – Pearson, green – Spearman) at feature (gene expression) level between different ML methods: figures above the main diagonal – with FloWPS, figures below – without FloWPS.


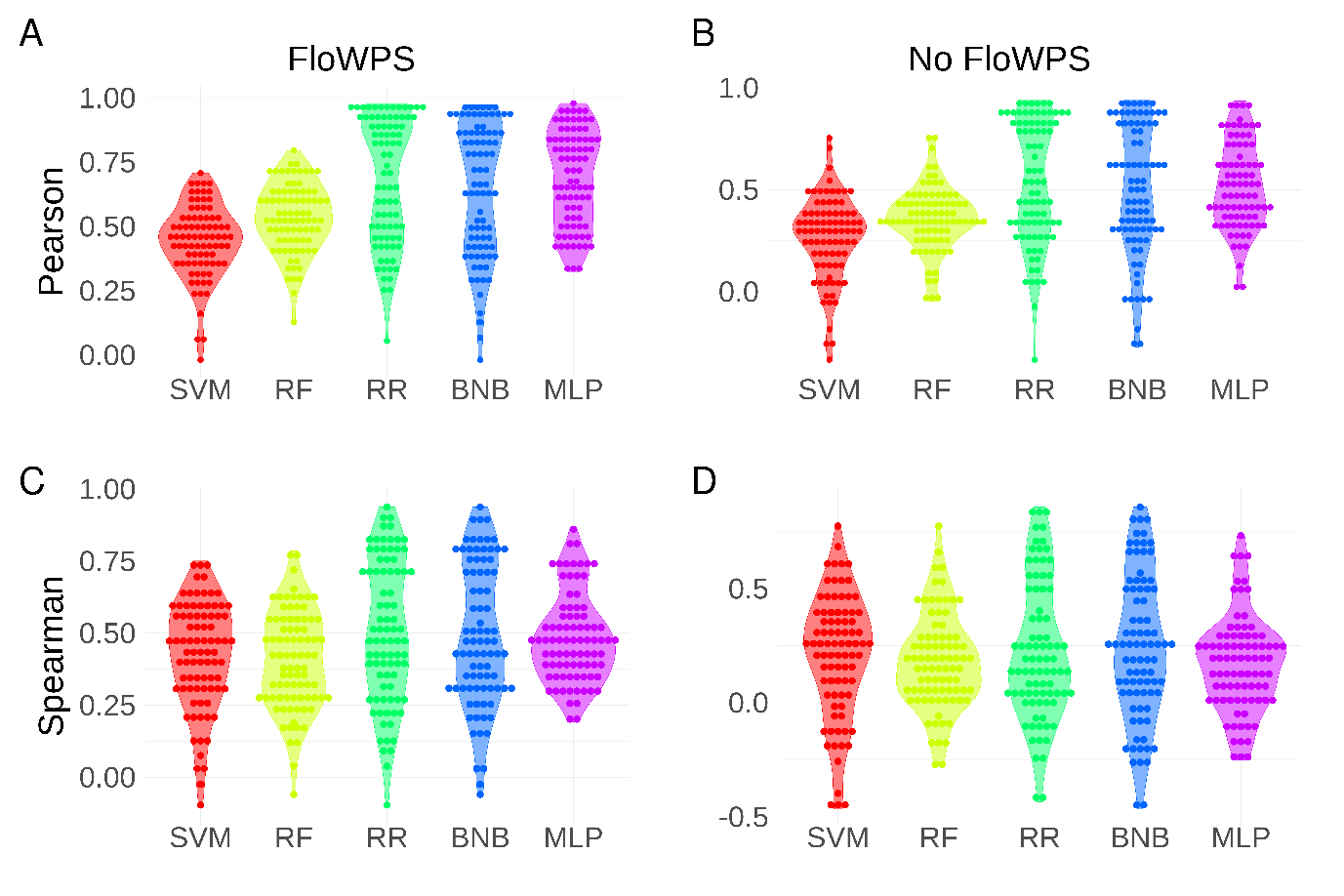


Fig. S3_2. Overall pairwise correlations as similarity metric for each ML method at feature (gene expression) level: A – Pearson, FloWPS, B – Pearson, no FloWPS, C – Spearman, FloWPS, D – Spearman, no FloWPS.
