# Supplementary material for: Flexible Data Trimming Improves Performance of Global Machine Learning Methods in Omics-Based Personalized Oncology
Source: Int J Mol Sci. 2020 Jan 22;21(3):713. doi: 10.3390/ijms21030713 (PMC7037338; doi:10.3390/ijms21030713)
Supplement: Supplementary file 1 [file ijms-21-00713-s001.zip › Suppl_1.docx]

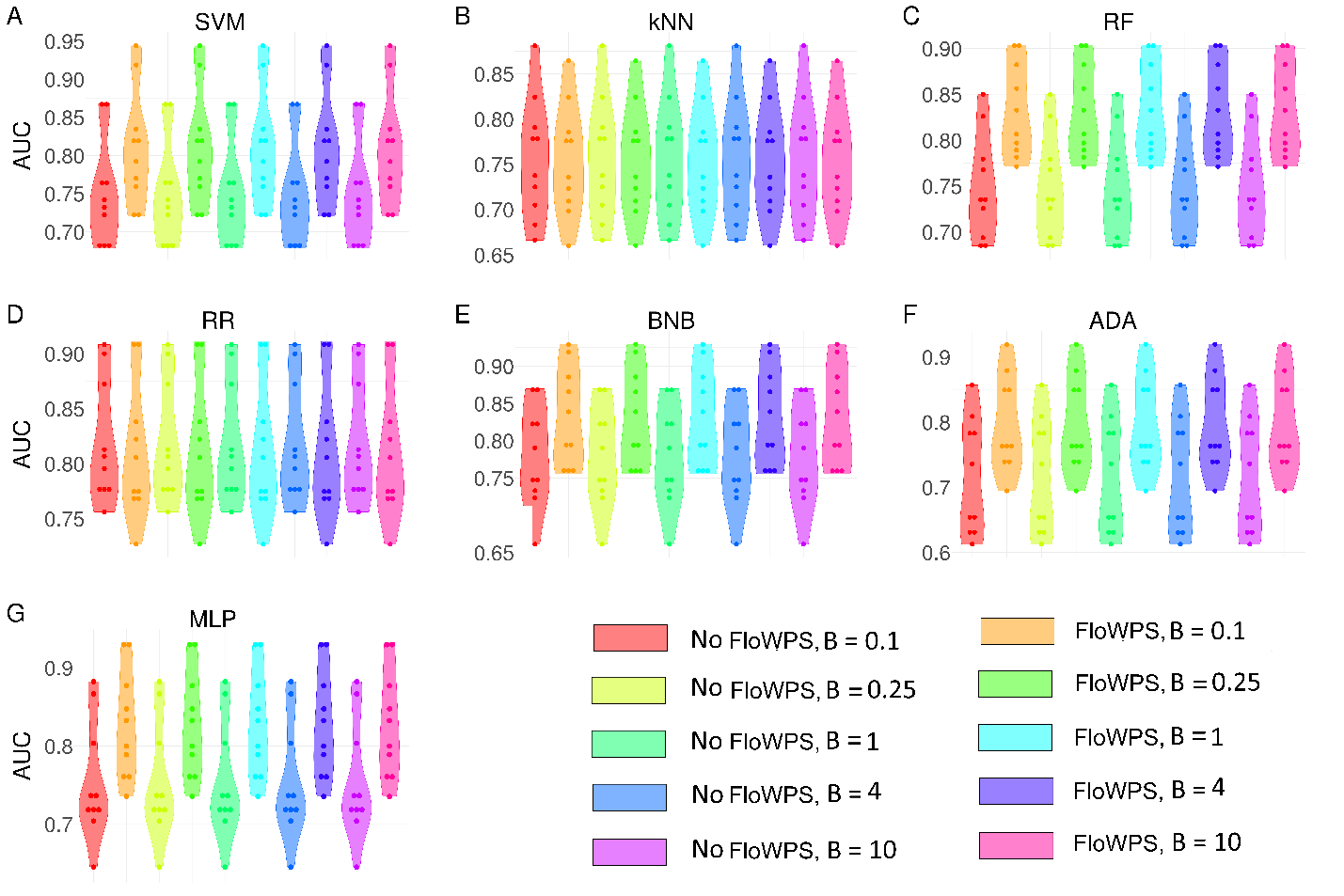


Fig. S1_1. Area under receiver-operator curve (ROC AUC) for treatment response classifiers for ten cancer datasets (see Table 1). The classifiers were based on SVM (A), kNN (B), RF (C), RR (D), BNB (E), ADA (F) and MLP (G) with default in-built parameter settings according to the Python package *sklearn* (Pedregosa et al., 2012). The color legend shows absence or presence of FloWPS in the classifier analytic pipeline and the value of relative balance factor *B*. On each panel, each violin plot shows distribution of values for eleven cancer datasets.


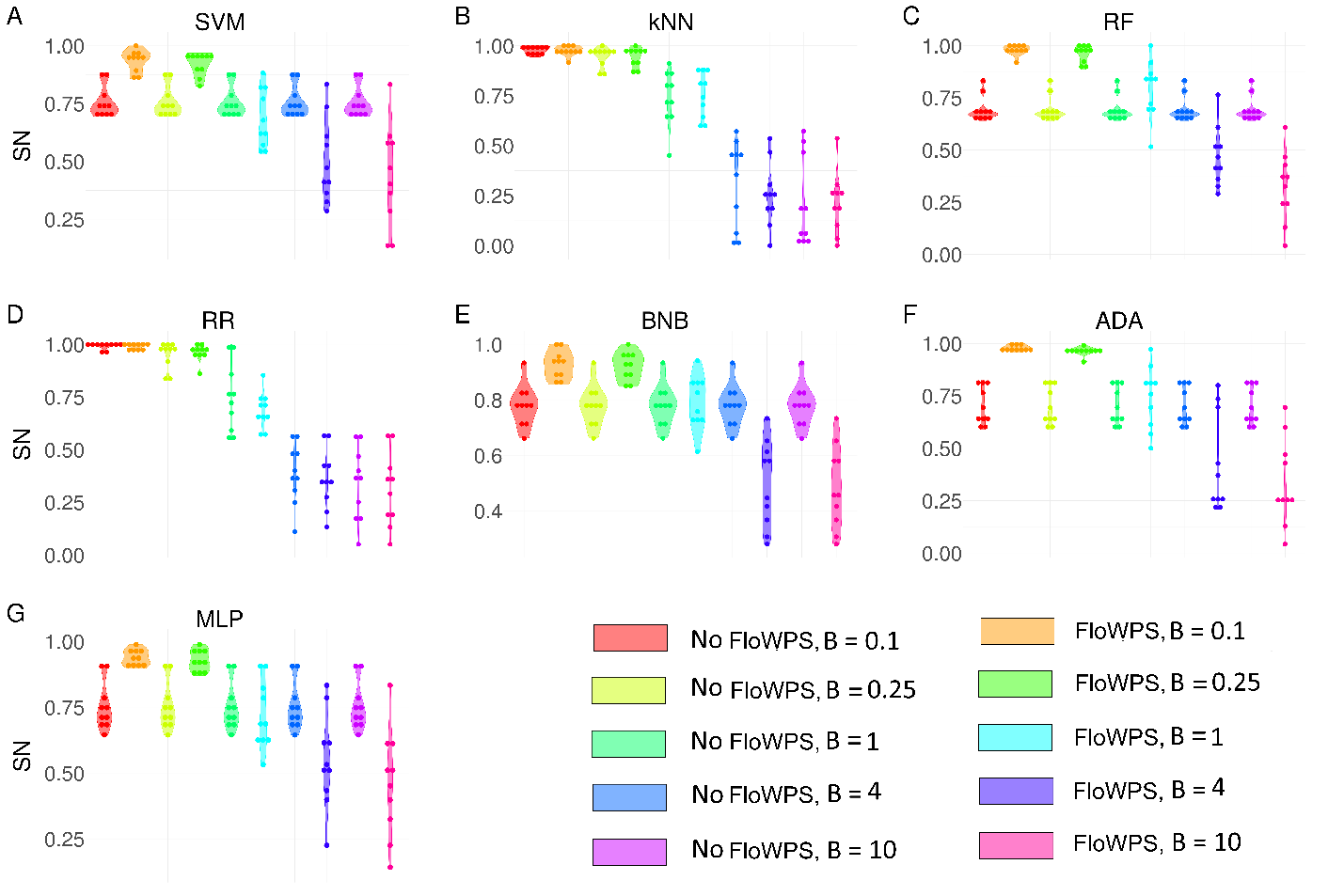


Fig. S1_2. Sensitivity (SN) for treatment response classifiers for ten cancer datasets (see Table 1). The classifiers were based on SVM (A), kNN (B), RF (C), RR (D), BNB (E), ADA (F) and MLP (G) with default in-built parameter settings according to the Python package *sklearn* (Pedregosa et al., 2012). The color legend shows absence or presence of FloWPS in the classifier analytic pipeline and the value of relative balance factor *B*. On each panel, each violin plot shows distribution of values for eleven cancer datasets.


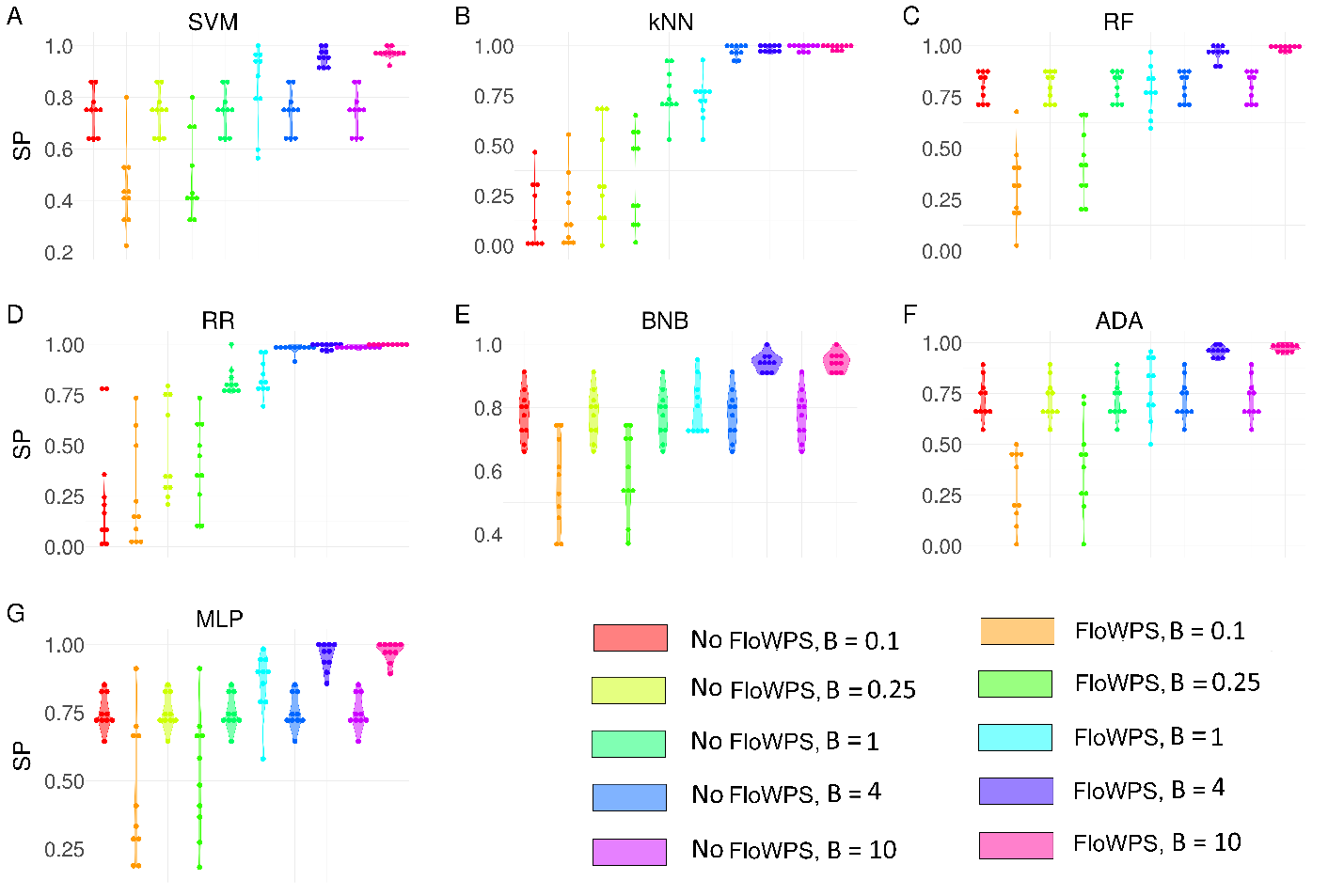


Fig. S1_3. Specificity (SP) for treatment response classifiers for ten cancer datasets (see Table 1). The classifiers were based on SVM (A), kNN (B), RF (C), RR (D), BNB (E), ADA (F) and MLP (G) with default in-built parameter settings according to the Python package *sklearn* (Pedregosa et al., 2012). The color legend shows absence or presence of FloWPS in the classifier analytic pipeline and the value of relative balance factor *B*. On each panel, each violin plot shows distribution of values for eleven cancer datasets.
